# Effects of Attitude, Barriers/Facilitators, and Visual Differentiation on Oral Mucosa Pressure Ulcer Prevention Performance Intention

**DOI:** 10.3390/healthcare9010076

**Published:** 2021-01-14

**Authors:** Min Kyeong Kang, Myoung Soo Kim

**Affiliations:** Department of Nursing, Pukyong National University, Busan 48513, Korea; ellekang1113@naver.com

**Keywords:** mouth mucosa, pressure ulcer, intention, attitude, primary prevention

## Abstract

Oral mucosa pressure ulcers (PUs) can result in frequent pain and discomfort, and have negative effects on quality of life. We aimed to examine attitude, barriers/facilitators of oral mucosa PU prevention, the ability to differentiate oral mucosa PU, and to identify factors influencing PU prevention performance intention. This was a cross-sectional descriptive study of 112 nurses in seven tertiary hospitals and three secondary hospitals. The data collection period was from August to December 2018. For data analysis, descriptive statistics, *t*-test, ANOVA, Pearson’s correlation coefficient, and multiple regression were used. The mean score of attitudes toward oral mucosa PU prevention was 3.74 ± 0.39. Barriers to oral mucosa PU prevention were 5.65 ± 1.66, and facilitators were 5.35 ± 1.34. The mean correct answer rate of visual differentiation ability was 13%. The factors affecting intention to perform oral mucosa PU prevention were facilitators of oral mucosa PU prevention (β = 0.32, *p* = 0.001) and attitude (β = 0.26, *p* = 0.005). To increase intention to perform oral mucosa PU prevention, positive attitudes and enhanced facilitators should be encouraged. Therefore, standardized guidelines and strategies, such as educational opportunities and allocation of resources and personnel focused on oral mucosa PU prevention, need to be provided.

## 1. Introduction

Medical device-related pressure ulcers (MDRPUs) are defined as localized damage to the skin and underlying tissues caused by the use of medical devices [1]. The incidence rate of hospital-acquired pressure ulcers (PUs), including MDRPUs, is 41.2% [2]. The MDRPUs most commonly encountered by nurses are endotracheal tube (ETT)-related PUs, which account for 27.6% of MDRPUs [3]. Additionally, 45.0% of oral mucosa PUs are caused by fixation devices or the ETT itself [4]. PUs in the lower lip have been reported at a higher frequency than those in the upper lip [5]. Oral mucosa PUs can result in frequent pain and discomfort [6], and have negative effects on health-related quality of life, as they can cause deterioration in the systemic infection related to ventilator-associated pneumonia [7]. Therefore, this issue warrants greater attention, which must include frequent assessment, periodic relocation, and early removal of the ETT for the prevention, and early detection, of oral mucosa PUs [8]. However, there seems to be a low interest in oral health among nurses. A study reported that only 44% of qualified oncology nurses documented oral an assessment merely once per day [9]. Moreover, as the performance rate of general PU prevention is as low as 67.6% [10], that of oral mucosa PUs would be even lower. Even though several strategies, including clinician self-report, patient-report, and medical record review are recommended for checking preventive care performance, their evidence-based effectiveness remains limited [11].

Identifying a person’s intention is an effective way to predict their actual behavior [12]. Performance intention represents one’s self-awareness of one’s plan to perform an action [13], and is an effective self-regulatory strategy to facilitate pre-planned actions [14]. In a previous study, the group which had higher acceptance intention regarding a smartphone healthcare application tended to perform health-promoting behaviors [15]. Furthermore, performance intention had a direct effect on actual PU prevention performance, which explained 52.7% of the variance [16]. Therefore, it is hypothesized that performance of oral mucosa PU prevention can be predicted by identifying the performance intention of nurses. 

The intentions were determined by three factors, including attitude, subjective norm, and perceived behavioral control [17]. Having a positive attitude can increase the likelihood of behavioral change [18], and is positively related to performance [19]. Therefore, negative attitudes toward PU prevention may negatively affect preventive care performance [20]. Furthermore, an individual’s attitude has a direct impact on PU prevention performance intention [16], and is regarded as an outcome variable, similar to performance intention [21]. However, a positive attitude alone is not sufficient to ensure PU prevention, and other factors should also be considered alongside it [22]. Particularly, barriers such as limited staff knowledge and physical skills [23], work overload due to understaffing, communication strategies, clarity of roles and responsibilities [24], and lack of resources [25] often limit adherence to PU prevention guidelines. In addition, teamwork, effective communication [24], and positive beliefs about consequences or capabilities are known to be facilitators of PU prevention [23]. Based on the fact that the “fear of adverse consequences” might facilitate positive attitudes and motivate nurses to prevent PUs [23], a multifaceted approach is required to take into account the barriers and facilitators intrinsic to the organizational context.

It is important to precisely assess and distinguish PUs [26], as it is a crucial competence for PU preventive care providers [25]. However, to date, there has been a paucity of research evaluating healthcare workers’ competence in visual differentiation using photographs [26]. There is insufficient photographic evidence of oral mucosa PUs [27], which makes it more challenging to evaluate the discrimination ability of healthcare providers. Owing to their poor visibility, early detection of oral mucosa PUs is also difficult. Assessing the impact of visual differentiation ability on performance intention could provide more extensive information [26] than narrative knowledge using a paper-based survey. Furthermore, most recent empirical studies have treated barriers and facilitators of perceived behavioral control as both direct determinants of intention, and interaction variables [27,28]. If the effects of the interaction between attitude, and barriers or facilitators of oral mucosa PU prevention performance intention, are identified, it may indirectly prove the moderating role of barriers and facilitators in the relationship between attitude and intention; and this may further help refine guidelines for intubated patients. Therefore, the current study sought to identify the factors influencing oral mucosa PU prevention performance intention.

## 2. Materials and Methods

### 2.1. Design

A cross-sectional descriptive study design was used to identify the factors affecting oral mucosa PU prevention performance intention.

### 2.2. Participants and Data Collection

Purposive sampling was employed to recruit registered nurses who have taken care of intubated patients working in intensive care units (ICUs), medical/surgical wards, and anesthesiology departments at seven tertiary hospitals and three secondary hospitals in Korea. The inclusion criterion for participating registered nurses (RNs) was having at least three months of clinical experience. We excluded administrative nurses, such as unit managers or charge nurses from the study. The required number of participants was calculated using the G*Power 3.1.9.2 analysis program. Cohen’s f^2^ input variables were used to calculate the effect size, and consequently for the multiple regression, which was estimated using R^2^, based on a previous study [29]. Considering a 10% drop-out rate because of missing data, 120 questionnaires were distributed to staff nurses. Respondents placed the completed, sealed questionnaires in a box located in the nurse station on each floor, and the research assistants collected the questionnaires. In total, 116 questionnaires were returned (response rate of 96.7%) and 112 were finally analyzed, after excluding four questionnaires having missing answers. Data were collected from August to December 2018.

### 2.3. Instrument

#### 2.3.1. General Characteristics

General characteristics consisted of eight items: age, gender, marital status, educational level, working department, total clinical experience, clinical experience in the current department, and number of beds. 

#### 2.3.2. Intention to Perform Oral Mucosa PU Prevention

Items for intention to perform general PU prevention developed by Lee, Park, and Park [16] were modified in accordance with the needs of this study. The questions required modification because the nurses’ interest in the development of oral mucosa PUs and the application of preventive care did not seem to be high. In this study, we extracted two questions that measured the intention to conduct prevention mainly by presenting situations related to compliance with guidelines or regulations in the medical/surgical, emergency/regular, or potential/actual context [16,30]. For example, “I try to comply with the injury prevention regulations when caring for patients with a high risk of oral mucosa PUs” and “In order to improve nursing care quality, I try to follow the injury prevention regulations for oral mucosa PU prevention.” Two wound nurses and a nursing professor examined the content validity. The item-level content validity index (I-CVI) and scale-level content validity index (S-CVI) were both found to be 1. These were acceptable based on the guideline that I-CVI should be 1.00 when there are five or fewer experts [31], and S-CVI should exceed 0.9 [32]. The previously reported Cronbach’s alpha of this instrument was 0.87 [16], and it was 0.88 for this study. Each question was measured on a five-point Likert scale from 1 (not at all) to 5 (always). The mean score of the items was used for analysis. A higher score indicated a positive intention to perform oral mucosa PU prevention. 

#### 2.3.3. Attitude

To examine the attitude toward oral mucosa PU prevention, we used an 11-item questionnaire developed by Moore and Price [25] and verified in Korean by Seo [33]. After modifying the wording from PU to oral mucosa PU, I-CVI and S-CVI were calculated by two wound nurses and one nursing professor using a four-point scale (4 = highly relevant, 3 = quite relevant, 2 = somewhat relevant, 1 = not relevant). I-CVI and S-CVI were found to be acceptable. We employed a five-point Likert scale (from 1 = definitely disagree to 5 = definitely agree). A high score indicated a positive attitude toward oral mucosa PU prevention. Cronbach’s alpha in a recent study was 0.79 [33], and for this study was 0.70. Compared to the previous study, the reliability of this study may have been lower for two reasons: first, the respondents may have had a problem with the questions because of their unfamiliarity with the meaning of oral mucosa PU; second, they may not have been aware of the “potential risks” because of limited information about the risk factors of oral mucosa PUs. However, internal consistency in this study can be considered appropriate for analysis, because the cut-off for acceptable reliability is generally considered to be 0.70 and above in the field of social science [34].

#### 2.3.4. Barriers and Facilitators of Oral Mucosa PU Prevention

Barriers and facilitators from the oral mucosa PU prevention scale developed by Kim and Ryu [21] were employed after modification. The themes of the barriers include seven items, and those of facilitators include six items. We employed a numeric rating scale from 0 (not at all) to 10 (always). Cronbach’s alphas in a previous study were 0.81 for barriers, and 0.78 for facilitators [21]. In this study, Cronbach’s alphas for barriers and facilitators were 0.92 and 0.88, respectively.

#### 2.3.5. Visual Differentiation Ability

To test the visual differentiation ability of the nurses for oral mucosa PUs, nine photographs [5], derived from a prospective observational study were used. First, 82 photographs were obtained from data from 113 patient-days of 17 patients in the ICU, and their content validity was evaluated by one wound nurse, one ICU nurse, and one dental surgeon, with a four-point scale based on the modified Reaper oral mucosa pressure injury scale (ROMPIS) [34] (stage 0 = normal, stage 1 = redness of mucosa/demarcation of mucosa/non-blanchable erythema; stage 2 = destruction of mucosa/soft coagulum or clotting on the mucosa/damage to the epidermal and dermal layers; stage 3 = damage to the fascia/exposure of muscle). Only 17 photographs with an appropriate I-CVI were selected. Second, two dentists selected nine valid photographs, excluding eight photographs with low resolution and brightness. There were three photographs of stage 0, two of stage 1, three of stage 2, and one of stomatitis. There were no photographs of stage 3 in the original data, literature, and online data. However, we still included the description of stage 3 in the questionnaire because its inclusion seemed necessary to help introduce the participants to the stage system of oral mucosa PUs. Five wound and ostomy care nurses were asked to identify nine photographs. Krippendorff’s alpha coefficient, as a measure of inter-rater reliability, was 0.75 (95% CI = 46–85). The participants were asked to choose one of the six options: stage 0 to 3, not any stage, and “I don’t know.” The answers were classified as correct (coded 1) or incorrect (coded 0). The possible range of the mean score was from 0 to 1, where a higher score was indicative of a higher visual discrimination ability. Internal consistency of this study was 0.70.

### 2.4. Ethical Considerations

This study was approved by the institutional review board (1041386-20180614-HR-017-03). During the recruitment period, the purpose, voluntary nature of participation, confidentiality of information, and procedures of the study were explained to the nurses. Informed consent was obtained from each nurse.

### 2.5. Data Analysis

All statistical analyses were performed using IBM SPSS for WIN (SPSS, Inc., Chicago, IL, USA). Descriptive statistics, independent *t*-tests, and one-way analyses of variance with Scheffé post hoc test were used to describe the intention to perform oral mucosa PU prevention according to the participants’ characteristics. Pearson’s correlation coefficient was used to define the relationships between the research variables. For performing multiple regression, evaluation results of assumptions, including normality, linearity, and homogeneity at the univariate and multivariate levels, or of bivariate scatterplots between pairs of variables were satisfactory [29]. Furthermore, the absence of multicollinearity and singularity was checked. Standard multiple regression analysis was performed to examine the factors influencing performance intention, including the main and interaction effects. Based on the univariate analysis, we identified three significant independent variables (attitude, barriers, and facilitators) and three interaction terms (attitude*barriers, attitude*facilitators, and barriers*facilitators). Before conducting the analysis, “centering” was conducted for the interaction terms of the independent variables to decrease multicollinearity, by subtracting the participants’ score from the mean score of each independent variable.

## 3. Results

### 3.1. General Characteristics of the Participants

Almost all of the participants (95.5%, *n* = 107) were women. The mean age was 31.16 years, 77.7% (*n* = 87) of the participants were unmarried, and 84.0% (*n* = 94) of them had a bachelor of science degree in nursing. About half of the participants worked in ICUs (49.1%, *n* = 55), and 50.9% (*n* = 57) of the participants had 1–5 years of total clinical experience. There were significant differences in the intention to perform oral mucosa PU prevention among the working departments of the nurses (F = 3.66, *p* = 0.029), specifically, participants working in ICUs had higher scores (3.82 ± 0.64), compared to those working in medical/surgical wards (3.50 ± 0.62) (see Table 1).

### 3.2. Characteristics of the Variables

The nurses’ mean scores of attitudes, barriers, and facilitators of oral mucosa PU prevention, and visual differentiation ability were 3.74 ± 0.39, 5.65 ± 1.66, 5.35 ± 1.34, and 0.13 ± 0.13, respectively (see Table 2). The mean score of performance was 3.68 ± 0.65. Among the barriers, the item with the highest score was “There is insufficient expert provision for preventing oral mucosa PUs” (6.49 ± 2.27), and the item with the lowest score was “My competence in oral mucosa PU prevention is insufficient” (4.72 ± 2.07). Among the facilitators, the item with the highest score was “Preventing oral mucosa PUs can also prevent most PUs” (6.41 ± 1.74), and the item with the lowest score was “Education opportunities for PU prevention are sufficient” (3.88 ± 1.87) (see Table 3). 

### 3.3. Correlation between Variables

Intention to perform oral mucosa PU prevention was positively associated with attitude (r = 0.38, *p* < 0.001) and facilitators (r = 0.43, *p* < 0.001), and was negatively associated with barriers (r = −0.33, *p* < 0.001); however, it had no significant relationship with visual differentiation ability (r = −0.10, *p* = 0.273). Attitude toward oral mucosa PU prevention was negatively correlated with barriers to oral mucosa PU prevention (r = −0.36, *p* < 0.001), and positively correlated with facilitators of oral mucosa PU prevention (r = 0.39, *p* < 0.001). (see Table 4).

### 3.4. Factors Affecting Intention to Perform Oral Mucosa PU Prevention

**Figure 1:**
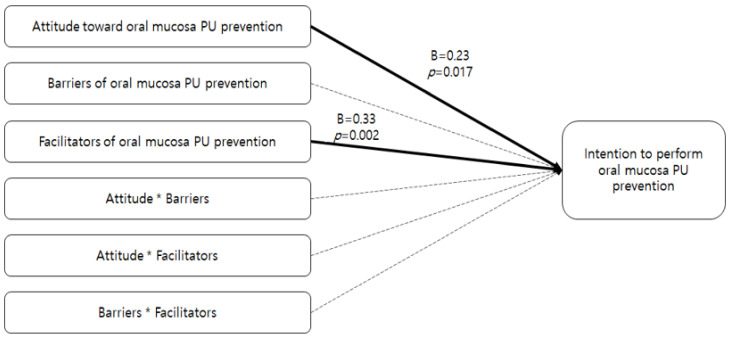
Diagram for results of multiple regression.

Assumptions were tested before the multiple regression analysis. The tolerance ranged from 0.23 to 0.78, and the variance inflation factor (VIF) ranged from 1.29 to 4.27. The VIF value was lower than 10, and the tolerance was higher than 0.2 based on the recommended criteria [35], so there were no issues related to multicollinearity. The Durbin–Watson statistic was close to 2 at 2.022, indicating that there were no issues related to autocorrelation. It also met the assumptions about the residuals regarding equal variances and normal distributions. Table 5 shows the factors influencing intention to perform oral mucosa PU prevention. The influencing factors were facilitators (β = 0.33 *p* = 0.002) and attitude (β = 0.23, *p* = 0.017), which explained 21.8% of the variance (F = 16.82, *p* < 0.001). There were no interaction effects (see Table 5, Figure 1).

## 4. Discussion

Although several studies have examined attitudes [36], as well as perceived barriers and facilitators, to performing preventive care of PUs [23,24,37], there are gaps related to the empirical study of oral mucosa PUs. Considering the high incidence rate of oral mucosa PUs [3,4,5,38], it would be helpful to define the factors influencing the intention to perform oral mucosa PU prevention. Therefore, based on the current study’s results, we focused on the significant factors for oral mucosa PU prevention performance intention. 

The mean score of intention to perform oral mucosa PU prevention was 3.68, which was similar to that of 3.57 for general PUs [16], or 3.80 for fall prevention intentions [39]. This showed that oral mucosa PUs were perceived as a crucial concern for intubated patients, though oral mucosa PUs have not yet been officially and clinically classified [40]. Considering general characteristics, ICU nurses had a higher performance intention than nurses in medical/surgical wards. This was consistent with a previous study that showed nurses in ICUs performed PU prevention more frequently than those in surgical wards, as their performance intention had a direct impact on actual performance [10]. ICU nurses might perceive their patients as a high-risk group for MDRPUs, and have a higher performance intention, because they usually take care of more intubated patients than nurses in wards [3]. 

The mean score of attitudes toward oral mucosa PU prevention was similar to that of skin PU prevention in previous studies [19,21]. According to recent studies, oral mucosa PU is a common MDRPU [4], it improves in 2–3 days, and relapses frequently compared to skin PUs [5]. This shows that nurses perceive oral mucosa PUs as an important health problem. In the current study, the mean scores of barriers and facilitators of oral mucosa PU prevention (barriers = 5.65, facilitators = 5.35) were higher and lower, respectively, compared with those observed in a previous study (barriers = 5.03, facilitators = 5.87) [21]. The mean score of visual differentiation ability related to oral mucosa PUs was 0.13, which was markedly lower than that of skin PUs in previous studies, which ranged from 0.32 to 0.55 [26,41], and that of narrative knowledge related to skin PUs, which ranged from 0.53 to 0.81 [26]. Although brief descriptions of the characteristics of oral mucosa PU stages were provided in the questionnaire, respondents might not have selected the correct answers for two reasons. First, the oral mucosa consists of two layers, which are the stratified squamous epithelium and the deeper lamina propria, contrary to the three skin layers [42]. These histological differences make it difficult to discriminate between the oral mucosa PU stages. Second, the nurses might be unfamiliar with identifying PU stages using photographs, instead of using narrative questionnaires. In addition, the stage classification system or photographic data of oral mucosa PUs, which could improve communication among clinicians and monitoring of oral mucosa PUs [34], remains undeveloped. Therefore, to improve the visual differentiation ability of nurses, the mucosal PU stage system, based on the characteristics of histological differences and healing of the mucous membrane [43], needs to be officially developed and used as a visual material [41].

Factors influencing performance intention were attitude and facilitators. This is in line with the findings that attitude affects intention to perform skin PU prevention [16] and actual performance [19]. Previous research has also shown a positive relationship between facilitators and performance, which corroborates the current study’s results [21]. Attitude was defined as behavioral intent and the amount of regard for or against an object, which is affected by knowledge [13]. This might be influenced by two factors, specifically, the nurses’ formal training in PU prevention [25], and their nursing priorities in the clinical setting [25]. Considering how frequently nurses deal with intubated patients [5], they need to be provided with formal training and information on prioritization for oral mucosa PU prevention. Therefore, educational programs in nursing should include a definition of oral mucosa PUs and a care algorithm for the same. The importance of oral mucosa PU prevention needs to be emphasized [44].

Based on the item analysis of facilitators, the scores relating to educational opportunities available to the nurses and communication among staff were low. Based on a previous study [24], availability of educational opportunities, effective cooperation and communication with the medical staff, and proper support for resources and personnel would be important factors to improve oral mucosa PU prevention performance. Therefore, three strategies should be prepared. First, educational opportunities, including regular brief training programs, need to be provided. Brief information, including the fact that oral mucosa PUs are highly affected by mechanical factors, such as the ETT itself and commercial ETT holder use [4], will greatly increase the likelihood of nurses repositioning the ETT and reducing the use of commercial ETT holders. Second, communication and teamwork training within the multidisciplinary team could be critical facilitators in the prevention of PUs [23,24]. Especially, because formal communication occurs in various forms, such as handover and documentation [21], quality improvement of PU preventive documentation needs to be continued. Third, appropriate RN staffing levels for daily care ensuring patient safety should be implemented. All nursing staff personnel, including RN and nurse aid staff, can contribute to the prevention of hospital-acquired PUs [45].

A recent study identified perceived behavior control not only had a direct effect on intention, but also interacted with other determinants of intention [46]. However, this study did not find any interaction effects on intention, which is different from the findings of previous studies. This may be owing to the relatively higher influence of attitude on oral mucosa PU prevention performance than on intention. Meanwhile, facilitators of oral mucosa PU prevention may lead to higher intention. Based on the theory of planned behavior, a favorable attitude provides the motivation to perform the behavior; however, an intention becomes more concrete only when perceived control over the behavior is strong [17]. It is necessary to derive more stable results by performing repeated studies, as it is difficult to predict the effect of the interaction between attitude and perceived behavioral control on oral mucosa PU prevention performance intention based on this study.

This study has yielded useful data on the factors influencing intention to perform oral mucosa PU prevention. However, it has a number of limitations. First, other factors influencing performance intention, such as anticipated affect, past behavior [47], interest in PU care [48], teamwork, communication, and commitment [49], were not included as input variables. Furthermore, only four factors, namely, attitude, barriers, facilitators, and visual differentiation ability, were considered as independent variables, without considering the participants’ characteristics. Second, when measuring attitudes, we simply replaced general PU with oral mucosa PU, which may have reduced the reliability of the instrument. Therefore, more discursive influencing factors and a more adequate instrument for evaluating intention to perform oral mucosa PU prevention would rectify these limitations.

## 5. Conclusions

This study has highlighted the factors affecting the intention to perform oral mucosa PU prevention, and provided basic data for enhancing the performance of oral mucosa PU prevention. Positive attitude among nurses and facilitators of oral mucosa PU prevention are expected to improve the intention to perform oral mucosa PUs, which may further improve actual performance. This suggests that a wider range of influencing factors need to be discovered in the future, and that educational programs and algorithms for oral mucosa PU prevention should be developed.

## Figures and Tables

**Table 1:** Differences in intention to perform oral mucosa PU prevention according to general characteristics of the participants (N = 112).

Characteristics	Categories	*n* (%)	Intention to Perform Oral Mucosa Pressure Ulcer (PU) Prevention	
			M ± SD	t/F (*p*)
Gender	Male	5 (4.5)	4.20 ± 0.89	1.85 (0.068)
	Female	107 (95.5)	3.65 ± 0.65	
Age (in years)	<30	60 (53.6)	3.65 ± 0.67	1.08 (0.362)
(M ± SD = 31.16 ± 6.06)	30∼39	40 (35.7)	3.69 ± 0.66	
	40∼49	9 (8.0)	3.61 ± 0.49	
	50 ≦	3 (2.7)	4.33 ± 0.58	
Marital	Married	25 (22.3)	3.86 ± 0.55	1.59 (0.116)
status	Single	87 (77.7)	3.63 ± 0.67	
Educational	College	9 (8.0)	3.61 ± 0.49	0.67 (0.935)
level	University	94 (84.0)	3.68 ± 0.68	
	Master ≦	9 (8.0)	3.72 ± 0.57	
Working	ICU	55 (49.1)	3.82 ± 0.64^ⓐ^	3.66 (0.029)
department	Medical-surgical ward	51 (45.5)	3.50 ± 0.62^ⓑ^	ⓐ > ⓑ
	Anesthesiology	6 (5.4)	3.83 ± 0.75	
Total clinical	1∼5	57 (50.9)	3.60 ± 0.67	0.48 (0.753)
experience	6∼10	39 (34.8)	3.73 ± 0.63	
(in years)	11∼15	5 (4.5)	3.80 ± 0.84	
(M ± SD = 7.11 ± 5.52)	16∼20	5 (4.5)	3.70 ± 0.67	
	20 ≦	6 (5.3)	3.91 ± 0.66	
Clinical experience	≦1	33 (29.5)	3.65 ± 0.63	1.02 (0.388)
in the current	2∼4	45 (40.2)	3.62 ± 0.71	
department (in years)	5∼9	23 (20.5)	3.67 ± 0.56	
(M ± SD = 4.25 ± 4.70)	10 ≦	11 (9.8)	4.00 ± 0.67	
Number of beds	<500	18 (16.1)	3.69 ± 0.67	0.01 (1.000)
	500∼<1000	46 (41.1)	3.68 ± 0.73	
	1000∼<1500	33 (29.4)	3.67 ± 0.52	
	1500 ≦	15 (13.4)	3.67 ± 0.70	

ICU = Intensive Care Unit; M = mean; SD = standard deviation; ^ⓐ,ⓑ^ analyzed using Scheffé post hoc test

**Table 2:** Descriptive statistics of study variables (N = 112).

Variables	M ± SD	Actual Range	Potential Range
Intention to perform oral mucosa PU prevention	3.68 ± 0.65	2.00–5.00	1.00–5.00
Attitude toward oral mucosa PU prevention	3.74 ± 0.39	2.90–4.70	1.00–5.00
Barriers of oral mucosa PU prevention	5.65 ± 1.66	0.57–9.43	0.00–10.00
Facilitators of oral mucosa PU prevention	5.35 ± 1.34	1.50–10.00	0.00–10.00
Visual differentiation ability for oral mucosa PU	0.13 ± 0.13	0.00–0.64	0.00–1.00

**Table 3:** Perceived barriers and facilitators of oral mucosa PU prevention (N = 112).

Category	Items	M ± SD
Barriers	My knowledge for of oral mucosa PU prevention is insufficient	5.42 ± 2.15
	My competence in oral mucosa PUs prevention is insufficient	4.72 ± 2.07
	There is not enough time to perform oral mucosa PU prevention	5.68 ± 2.21
	There is insufficient expert provision for preventing oral mucosa PUs	6.49 ± 2.27
	Priority given to preventing oral mucosa PUs is low	5.49 ± 2.06
	There are insufficient resources or tools to provide oral mucosa PU prevention	5.87 ± 2.18
	There are insufficient current record forms for oral mucosa PU risk factors or nursing interventions	5.85 ± 2.21
Facilitators	Education opportunities for oral mucosa PU prevention are sufficient	3.88 ± 1.87
	There is sufficient communication among staff about oral mucosa PU prevention performance	4.48 ± 1.97
	Preventing oral mucosa PUs can also prevent most PUs	6.41 ± 1.74
	The role of nurses in preventing oral mucosa PUs is clear	5.71 ± 1.88
	The organization is collaborative in performing oral mucosa PU prevention	5.67 ± 1.88
	My manager supports the implementation of oral mucosa PU prevention	5.93 ± 1.88

**Table 4:** Correlations among variables (N = 112).

Variables	1 r (*p*)	2 r (*p*)	3 r (*p*)	4 r (*p*)
1. Intention to perform oral mucosa PU prevention	1			
2. Attitude toward oral mucosa PU prevention	0.38(<0.001)	1		
3. Barriers of oral mucosa PU prevention	−0.33(<0.001)	−0.36(<0.001)	1	
4. Facilitators of oral mucosa PU prevention	0.43(<0.001)	0.39(<0.001)	−0.47(<0.001)	1
5. Visual differentiation ability for oral mucosa PU	−0.10(0.273)	−0.02(0.828)	0.10(0.308)	−0.17(0.079)

**Table 5:** Multiple regression on intention for performing oral mucosa PU care (N = 112).

Variables	B	SE	β	t	*p*	Tolerance	VIF
Attitude toward oral mucosa PU prevention (Attitude)	0.77	0.32	0.23	2.43	0.017	0.78	1.29
Barriers of oral mucosa PU prevention (Barriers)	−0.08	0.08	−0.10	−0.99	0.326	0.68	1.47
Facilitators of oral mucosa PU prevention (Facilitators)	0.32	0.10	0.33	3.12	0.002	0.64	1.57
Attitude*Barriers	0.17	0.21	0.11	0.80	0.425	0.35	2.83
Attitude*Facilitators	−0.07	0.06	−0.19	−1.09	0.277	0.23	4.27
Barriers*Facilitators	−0.24	0.27	−0.15	−0.15	0.367	0.25	3.99

R^2^ = 0.260, Adj. R^2^ = 0.218, F = 6.14, *p* < 0.001.

## Data Availability

Data sharing not applicable.
